# Non-pharmacological treatment of hypertension in primary health care: A comparative clinical trial of two education strategies in health and nutrition

**DOI:** 10.1186/1471-2458-11-637

**Published:** 2011-08-10

**Authors:** Amanda G Ribeiro, Sônia MR Ribeiro, Cristina MGC Dias, Andréia Q Ribeiro, Fátima AF Castro, Maria M Suárez-Varela, Rosângela MM Cotta

**Affiliations:** 1 Departamento de Nutrição e Saúde. Universidade Federal de Viçosa. Av. Peter Henry Rolfs, s.n. - Campus Universitário, 36570-000, Viçosa - MG - Brazil; 2 Unit of Public Health and Environmental Care, Department of Preventive Medicine, University of Valencia. Valencia, Spain; 3 CIBER - Epidemiology and Public Health (CB06/02/0045), Spain; 4 Research Foundation. University Hospital Dr. Peset. Valencia, Spain

## Abstract

**Background:**

Poor adherence to non-pharmacological treatment of hypertension represents a serious challenge for public health policies in several countries. This study was conducted to compare two intervention strategies regarding the adherence of adult women to dietary changes recommended for the treatment of hypertension in a community covered by Primary Health Care Unit.

**Methods:**

This study is a randomized controlled trial of a sample composed of 28 women with hypertension enrolled in the Primary Health Care Unit located in the urban area of southeastern Brazil. The participants were already undergoing treatment for hypertension but devoid of nutritional care; and were divided into two groups, each composed of 14 individuals, who received interventions that consisted of two different strategies of nutritional guidance: monthly health education workshops alone (Group 1) and combined with family orientation through home visits (Group 2). Adherence to nutritional guidelines was evaluated by dietary, anthropometric, clinical and serum biochemical parameters, measured before and after the interventions. Knowledge on control and risk of hypertension was also investigated. The study lasted five months.

**Results:**

Mean age was 55.6 (± 2.8) and 50.7 (± 6.5) in the groups 1 and 2, respectively. The home orientation strategy promoted greater adherence to dietary changes, leading to a statistically significant improvement in the clinical, anthropometric, biochemical and dietary parameters. The group 2 reduced the consumption of risk foods (p = 0.01), oil (p = 0.002) and sugar (p = 0.02), and decreased body mass index (-0.7 kg/m^2^; p = 0.01); waist circumference (-4.2 cm; p = 0.001), systolic blood pressure (-13 mm HG; p = 0.004) and glycemia (-18.9 mg/dl; p = 0. 01). In group 1 only waist circumference (-2 cm; p = 0.01) changed significantly.

**Conclusion:**

Nutritional orientations at the household level were more effective with regard to the adherence of individuals to non-pharmacological treatment of hypertension, regarding the reduction of clinical and behavioral risk parameters.

## Background

Cardiovascular disease accounts for one third of deaths worldwide. In the Americas, the World Health Organization reports about 140 million people suffer from hypertension (1). Hypertension is one of the most important risk factors of cardiovascular and kidney disease and is a serious public health problem. The estimated prevalence in Brazil is 35% of the population over the age of 40 years. There is a growing prevalence among the young population, representing approximately 17 million affected individuals (2).

Changes in lifestyle are fundamental to the prevention and control of hypertension, with diet figuring prominently. Since the 1970s, different countries have introduced programs of health promotion and the prevention of cardiovascular risk factors related mainly to diet, physical activity and quitting smoking (3). In Brazil, there has been an emphasis in recent decades on the importance of public policies directed at nutrition in the prevention of cardiovascular disease. The National Diet and Nutrition Policy (4) and the adoption of the Global Strategy on Healthy Eating, Physical Exercise and Health of the World Health Organization (WHO) (5) are examples of public policies that include recommendations regarding healthy eating as a way to control and prevent cardiovascular disease on the primary care level.

In Brazil, the Family Health Program (FHP) is a priority strategy of the Ministry of Health to organize primary care. It has proven to be an adequate model for addressing cardiovascular disease by means of prevention and health promotion aimed at changing the behavior and living habits of individuals without losing sight of the interactions in collective and social spheres (6,7).

Health education practices in primary health care have traditionally been guided by discourse focused on biological factors and hygiene, addressing disease and verticalized, prescriptive, information (7,8). This traditional education model has limits, as access to information alone does not determine a greater commitment on the part of hypertensive patients to their treatment (9). The adherence of such individuals to treatment is a complex, involving a multidimensional process influenced by multiple factors such as related to the individual, health status, therapy, socioeconomic aspects, the health care system and relationships with health care professionals (10).

The Family Health Program (FHP) is a health education model that is more coherent with the principles of integrality, respect and the autonomy of individuals. Its objectives are the humanization of health practices through the formation of ties between professionals and users, the democratization of knowledge on the health-illness process, the development of civic inclusion and the encouragement of the organization of the community (8). In this context, the home visit, which is part of the FHP routine, constitutes one form of holistic care, allowing an understanding of the psychological, emotional, social and biological aspects of users of the public health care system (11).

## Objectives

The aim of the present study was to compare two nutritional intervention modalities with regard to the adherence of individuals to dietary orientation indicated in the treatment of hypertension and to investigate their knowledge on the disease in a community covered by the FHP. The first intervention was based on group education activities held at the Primary Health Care Unit. The second modality combined the group education activities with home visits that followed a systematic nutritional education program and family follow up.

## Methods

This study was carried out at the Primary Health Care Unit in an urban area of the city, in Porto Firme, Minas Gerais State, Brazil, an area where, two Family Health teams cover a total of 1361 families and 626 individuals with hypertension. The choice of the city was due to the high FHP coverage, which is over 90%. Moreover, the FHP teams hold monthly group activities with individuals with hypertension, but devoid of guidance by nutritionists.

### Study Design

The study is a randomized (by lots), non-blinded trial involving 28 participants divided into two groups. Group 1 was made up of women who received orientation regarding the dietary treatment of hypertension through monthly dialogic education workshops, as mentioned above. Group 2 participated in the educative workshops and also received personalized orientation through periodic home visits.

**Figure 1:**
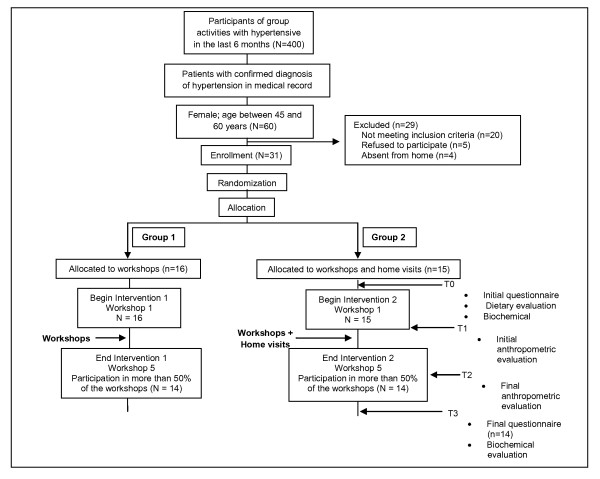
**Flowchart of the study involving women with arterial hypertension; Porto Firme, MG, Brazil, 2009**.

The final sample was made up of 28 women (14 in each group) covered by the Primary Health Care Unit who met the eligibility criteria, agreed to participate and attended at least two of the five nutritional education workshops (Figure 1).

### Participants

Women with hypertension and registered at Primary Health Care Unit were selected for the study. The inclusion criteria were: female gender; age between 45 and 60 years; with a clinical diagnosis of hypertension; and availability to participate in group activities. The following were the exclusion criteria: serious clinical condition with need for specialized care; chronic kidney disease; pregnancy; history of alcoholism or drug abuse. The individual clinical charts of the users at the Primary Health Care Unit were used for the determination of the eligibility criteria, with the assistance of the community health agents.

The recruitment of the sample was conducted in March 2009. In this period there have been screened 380 individuals who had participated in at least one group activity in the previous six months, according to information given by community health agents. There have been analyzed 276 medical records (122 records could not be found or were incomplete). Of a total of 276 individuals, 238 had a diagnosis of hypertension recorded in their medical profile. Of these, 159 were female, among which 60 were between 45 and 60 years, and 20 fell into some of the exclusion criteria.

After screening, there had been selected 40 participants who fulfill the inclusion criteria. The women have been approached in their homes and were informed about the objectives of the study. The women who were absent in more than three visits and the ones who refused to participate were excluded from the sample, totalizing nine women. After the randomization, three women have been excluded from the study for being present in less than 50% of the workshops. Finally, 28 women were selected. They have been allocated, randomly, in one of the two comparison groups.

The females were chosen because women, in the context of public health policies, stand out in their abilities of caregivers and also because they are more health aware, either for themselves, as for their relatives. The woman at home has a central role in the care of relatives in relation to hygiene, nutrition, treatment of diseased and self-care. Thus, they are the protagonist in the maintenance and transformation of dietary habits of families (12, 13).

The choice of this particularly age group is justified by the scientific and epidemiological knowledge accumulated, showing consistently higher prevalence of hypertension and its complications in individuals from middle age (14), setting a defining moment for delaying/preventing complications of diseases, increasing the chances of a healthy and active aging (15).

The study received approval from the Ethics Committee of the *Universidade Federal de Viçosa *(process number: 030/2009). All participants signed terms of informed consent.

### Intervention

**Figure 2:**
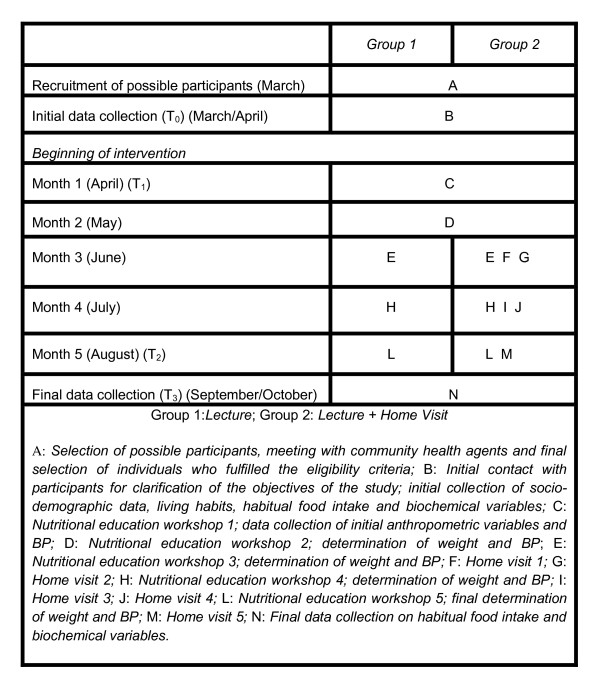
**Graphic representation of intervention involving women with arterial hypertension in Porto Firme, MG, Brazil (2009)**. Graphic representation of intervention involving women with arterial hypertension in Porto Firme, MG, Brazil; squares represent fixed elements, measurements and calculations; circles represent flexible activities; graphic method proposed by Perera et al., 2007 (32).

The intervention lasted five months and involved the following strategies and activities as described in Figure 2.

Five nutritional education workshops were held in different locations of the city in order to facilitate the access of the population. The workshops addressed topics on hypertension and dietary treatment measures through dialogic, dynamic, interactive lectures using posters, figures, videos and practical demonstrations.

Five home visits to each participant in Group 2 were carried out in order to establish systematic, but flexible orientation in accordance with the needs and conditions of each family. The visits lasted from 30 minutes to one hour. Thematic content of the workshops on health education and goals of home visits are outlined in Table 1.

**Table 1:** Thematic content of the workshops and home visits addressed in the education activities with hypertensive women in the city of Porto Firme - MG (2009).

Workshops	Home Visits
1 - Hypertension: concept, risk factors and dietary measures of treatment.	1 - Identification of excessive use of oil, salt and sugar and orientation about its correct use.
2 - Consumption of fat/oil, sugar and salt adding: recommendation and dangers of overconsumption.	2 - Identification of foodstuffs purchased at home and implementation of guidelines in accordance with the usual diet of the family.
3 - Consumption of fruits and vegetables: significance and recommendations.	3 - Strengthening guidance, answering questions and carrying out necessary adjustments according to the specific needs of each family.
4 - Brazilian Food Pyramid - fundamental concepts: proportionality, moderation and variety	4 - Strengthening guidance, answering questions and carrying out necessary adjustments according to the specific needs of each family.
5 - Physical activity: benefits and importance for weight reduction/maintenance.	5- Identification main changes in the feeding pattern of the participant and her family the achievements and setting goals to overcome.

### Adherence to nutritional orientation

The analysis of adherence was carried out through the evaluation of the following clinical and nutritional parameters before and after the intervention: weight, height, body mass index (BMI) and waist circumference, fasting glycemia, triglycerides, total cholesterol and cholesterol fractions, habitual food intake and blood pressure. The data on control and risk of hypertension were collected through a semi-structured questionnaire previously validated.

The anthropometric variables were measured during the nutritional education workshops (T1/T2), as proposed by Jellife (16). BMI was calculated as weight divided by height squared (Kg/m^2^). Waist circumference was determined using a non-extendable tape and measured in centimeters at the point of the smallest circumference.

Blood pressure (BP) was determined in accordance with the procedures recommended by the American Heart Association (17) using the routine nursing methods employed at the Primary Health Care Unit. Biochemical variables were analyzed using routine clinical methods and the participants were previously instructed to remain fasting for 12 hours prior to the blood collection. Habitual food consumption was assessed using a food intake frequency questionnaire validated in Brazil (18). Consumption of oil, salt and sugar were estimated from the monthly availability of these products divided by the days of the month and number of diners to home.

### Data analysis

Excel for Windows 2007 and SPSS for Windows (Version 18.0; SPSS Inc, Chicago, IL, USA) were used for the statistical analysis. Anthropometric, clinical, biochemical and food intake variables before and after the interventions were analyzed using the non-parametric Wilcoxon test, with the level of significance set at p < 0.05. The choice of this statistical test is appropriate for studies with small sample size and when the variables of interest do not show normal distribution (19). Data on knowledge and grasp of hypertension and per capita/day intake were analyzed using frequency distribution.

Regarding to the data on food intake, individual intake scores were calculated using the method proposed by Fornés *et al*. (20). The scores were obtained for six different intake frequencies for each food: f_1 _- non-consumed foods; f_2 _- foods consumed one to three times a month; f_3 _- foods consumed once a week; f_4 _- foods consumed two to four times a week; f_5 _- foods consumed five to six times a week; f_6 _- foods consumed daily. The highest score was attributed to foods consumed on a daily basis (S_6 _= 1). Foods were categorized into two groups: Class 1, with foods considered of risk (whole milk and byproducts, fried foods, foods rich in animal fat, sugar and salt); and Class 2, with protective or neutral foods, such as rice, beans, whole-grain cereals, fresh fish, olive oil, low-fat milk and byproducts, fruit, vegetables and legumes.

## Results

### Baseline characteristics of participants

In table 2 the socio-demographic and health characteristics of the 28 participants are summarized. There were no statistically significant differences between groups, except for the use of medications, which was higher in Group 2, and age, which was higher in Group 1.

**Table 2:** Socio-demographic and health characteristics of both groups of hypertensive women participating of the intervention strategies (Groups 1 and 2); Porto Firme, MG, Brazil, 2009.

	Group 1 (workshops) N = 14	Group 2 (workshops + visit) N = 14	z	p^a^
**Age in years (± SD)**	55.6 ± 2.8	50.7 ± 6.5	2.009	0.04*
**Schooling, years of study (± SD)**	3.9 ± 3.3	4.4 ± 3.2	0.658	0.51
**Regular use of medication for hypertension, % **	100	100	-	-
**n° medications/day (± SD)**	1.9 ± 0.6	2.8 ± 1.1	2.201	0.02*
**n° of pills/day (± SD)**	2.8 ± 1.5	4.5 ± 2.4	1.471	0.08
**Weight, kg (± SD)**	75.3 ± 17.4	77.7 ± 14.3	0.597	0.55
**BMI, kg/m^2 ^(± SD)**	31.3 ± 6.9	33.4 ± 5.6	1.424	0.15
**Excessive alcohol intake, % (n)**	0	0	-	-
**Smoking habit, % (n)**	7.1 (1)	0	-	-
**Physical activity, % (n)** **Active**	57.1 (8)	57.1 (8)	-	-

Values in cells are mean ± standard deviation; **^a ^**p-value for difference between groups by Mann-Whitney test; *p < 0.05.

Mean age was 55.6 (± 2.8) years in Group 1 and 50.7 (± 6.5) years in Group 2. Mean schooling was four years of study [3.9 (± 3.3) in Group 1 and 4.4 (± 3.2) in Group 2]. All participants made use of anti-hypertension therapy. The participants used an average 2.3 different types of anti-hypertension drugs (1.9 ± 0.6 in Group 1 and 2.8 ± 1.1 in Group 2), with a mean intake of 2.8 ± 1.5 and 4.5 ± 2.4 pills/day in Groups 1 and 2, respectively. In both groups, mean BMI characterized obesity (31.3 ± 6.9 in Group 1 and 33.4 ± 5.6 in Group 2). Alcohol and cigarette intake was not common; only one woman in Group 1 used to smoke. A large portion of the participants were classified as active (57.1% in both groups), using the international physical activity questionnaire summary report (21).

### Effect of interventions on knowledge about control and risk of hypertension

When asked about the care necessary to control hypertension, 100% of the participants cited the use of anti-hypertension therapy at T0 and T3. Healthy eating was the second most often cited item (85.7 and 64.3% in Groups 1 and 2, respectively, at T0 and 85.7% in both groups at T3). The practice of physical activity was cited by 50% of the participants in both groups at T0 and 84.6 and 92.9% in Groups 1 and 2, respectively, at T3. Moderation in the consumption of alcohol and quitting smoking were cited by only one participant at T0 and by 69.2 and 85.7% in Groups 1 and 2, respectively, at T3. Controlling nervousness/anger (leading a more tranquil life) was cited by 32.1% in both groups of the participants at T0 and by 69.2 and 85.7% in Groups 1 and 2, respectively, at T3 (Table 3).

**Table 3:** Care necessary to control hypertension cited by both groups of hypertensive women before and after intervention; Porto Firme, MG, Brazil, 2009.

	Group 1 (workshops)		Group 2 (workshops + home visits)	
	**Initial (T0)**	**Final (T3)**	**Initial (T0)**	**Final (T3)**
**Anti-hypertension therapy**	100%	100%	100%	100%
**Healthy eating**	85,7%	85,7%	64,3%	85,7%
**Physical activity**	50%	84,6%	50%	92,9%
**Moderation in the consumption of alcohol and quitting smoking**	7,1%	69,2%	0	85,7%
**Controlling nervousness/anger**	32,1%	69,2%	32,1%	85,7%
**Reduction salt intake**	92,8%	100%	92,8%	100%
**Reduction in the consumption of oils and fats**	70%	92,3%	70%	100%
**Consumption of fruit, vegetables and legumes**	42,9%	85,7%	28,6%	69,2%

Regarding care specifically related to eating, 92.8% of the participants cited reducing salt intake at T0 as a way to control blood pressure and this figure rose to 100% by T3. A reduction in the consumption of oils and fats was cited by approximately 70% of the participants at T0 and by 92.3 and 100% in Groups 1 and 2, respectively, at T3. The frequency of responses regarding the consumption of fruit, vegetables and legumes rose approximately 40% in both groups between T0 and T3 (Table 3).

With regard to health complications related to hypertension, heart attack and stroke were cited by 82% of the participants at T0 and approximately 90% at T3. Kidney disease was the third most often cited complication (14.3% of the participants at T0 and approximately 30% at T3).

### Effect of intervention on dietary variables

There were no statistically significant differences in intake scores in Class 1- *risk foods *(n = 14) before and after the intervention for either of the food groups analyzed (Group 1 - Z = 1.5; P = 0.13; Group 2 - Z = 1.4; p = 0.14) (Table 4). In Class 2- *protective or neutral foods *(n = 14), however, there was a significant increase in the intake of protective or neutral foods (Z = 2.5; P = 0.01). Analyzing the foods separately, there was a significant increase in no-fat and low-fat milk intake (Z = 2.6; P = 0.007) and whole-grain bread (Z = 2.0; P = 0.04) and a reduction in the consumption of pig skin/scraps (Z = 2.2; P = 0.02) and sweetened artificial juice (Z = 2.1; P = 0.03) in Group 1. In Group 2, there was a significant increase in the consumption of whole-grain cereals (Z = 3; P = 0.002).

**Table 4:** Dietary variables in both groups of hypertensive women before and after intervention; Porto Firme, MG, Brazil, 2009.

	Group 1 (workshops)				Group 2 (workshops + home visits)			
	**Initial (T0)**	**Final (T3)**	**Z **	**p^a^**	**Initial (T0)**	**Final (T3)**	**Z **	**p^a^**
**Score - Food Class I **	2.7 ± 1.5	2 ± 1.4	1.5	0.13	4 ± 1.7	3.1 ± 1	1.4	0.14
**Score - Food Class II**	6.1 ± 2.4	6.7 ± 2	1.3	0.16	5.5 ± 1.4	6.6 ± 1.5	2.5	0.01*
**Whole-grain cereals**	0.2 ± 0.3	0.2 ± 0.2	0.5	0.59	0.1	0.5 ± 0.3	3.0	0.002*
**Not-fat and low-fat milk**	0.3 ± 0.1	0.67 ± 0.3	2.6	0.007*	0.4 ± 0.3	0.4 ± 0.4	1.4	0.14
**Whole-grain bread**	0.2 ± 0.1	0.4 ± 0.1	2.0	0.04*	0.4 ± 0.2	0.3 ± 0.1	0.1	0.86
**Pig skin/scraps**	0.1	0.0	2.2	0.02*	0.3 ± 0.1	0	1.4	0.14
**Sweetened artificial juice**	0.3 ± 0.2	0.1	2.1	0.03*	0.3 ± 0.3	0.2 ± 0.2	1.1	0.25
**Per capita/day of oil**	41.8 ± 17.8	21.9 ± 13.7	2.5	0.01*	35.9 ± 16	21.4 ± 10.9	3.0	0.002*
**Per capita/day of sugar**	106.9 ± 67.5	85.8 ± 70.1	1.3	0.17	100 ± 47.9	75.4 ± 45	2.1	0.02*
**Per capita/day of salt**	11 ± 8.4	8.6 ± 5.6	1.6	0.10	10.6 ± 6.4	8.1 ± 2.6	1.1	0.27

Values in cells are mean ± standard deviation; **^a ^**p-value for difference in values before and after intervention in Groups 1 and 2 by the Wilcoxon test; *p < 0.05.

Regarding the consumption of oil, sugar and salt, there was a statistically significant difference between T0 and T3 in Group 1 only for the per capita/day consumption of oil (Z = 2.5; P = 0.01); at T0, 100% of Group 1 had values greater than those recommended by the Brazilian food pyramid (16 ml/day), whereas 36% had values below the maximal recommended value at T3 (22). In Group 2, there were significant differences between T0 and T3 in the per capita/day consumption of oil (n = 12; Z = 3; P = 0.002) and sugar (n = 11; Z = 2.1; P = 0.02), with a mean reduction of 14 mL in per capita/day consumption of oil (25% at T0 and 50% at T3 had values below the maximal recommended value), and a mean reduction of 27 g of sugar in the per capita/day consumption; however, the mean consumption was 75 g at T3, which is above the maximal recommended value of 56 g/day (23). The Wilcoxon test revealed no significant difference in salt intake in either group; however, there was a significant reduction in per capita/day intake among individuals who consumed more than 15 g of salt/day (n = 4) at T0 (Z = 1.81; P = 0.03), with a mean reduction of 9 ± 1.3 g in daily salt intake by T3.

### Effect of intervention on clinical, biochemical and anthropometric variables

Among all the parameters analyzed, there was a statistically significant difference between T0 and T3 in Group 1 for waist circumference (Z = 2.1; P = 0.017), with a mean reduction of 2 cm. In Group 2, there were statistically significant differences between evaluations with regard to weight, BMI, waist circumference, systolic BP and glucose, with a mean weight reduction of 1.7 kg (min. = 0; max. = 8 kg) (Z = 2.09; P = 0.018), mean reduction in BMI of 0.7 Kg/m^2 ^(Z = 2.06; P = 0.019), mean reduction in waist circumference of 4.2 cm (min. = 0; max. = 11 cm) (Z = 2.9; P = 0.001), mean reduction in systolic BP of 13 mmHg (Z = 2.6; P = 0.004) and mean reduction in fasting glycemia of 18.9 mg/dl (Z = 2.5; P = 0.01). Regarding fasting glycemia in Group 2, 46% of the women had high initial values (> 99 mg/dl), which were reduced to values within the ideal range at T3 according to recommendations of ADA (24) (Table 5).

**Table 5:** Anthropometric, clinical and biochemical variables in both groups of hypertensive women before and after intervention; Porto Firme, MG, Brazil, 2009.

	Group 1 (workshops)				Group 2 (workshops + home orientation)			
	**Initial (T0)**	**Final (T3)**	**Z **	**p^a^**	**Initial (T0)**	**Final (T3)**	**Z **	**p^a^**
**Weight, kg**	75.3 ± 17.4	75.4 ± 16.3	-0.126	0.452	77.7 ± 14.2	76 ± 13.1	-2.098	0.018*
**BMI, kg/m^^2^^**	31.3 ± 7	31.3 ± 6.3	-0.157	0.440	33.4 ± 5.7	32.7 ± 5.3	-2.062	0.019*
**WC, cm**	100.3 ± 14.9	98.5 ± 14.5	-2.100	0.017*	104 ± 12.2	99.7 ± 11.5	-2.986	0.001*
**Systolic BP, mmHg**	119.3 ± 14.9	117.4 ± 15.9	-0.637	0.261	129.3 ± 17	116.1 ± 9.6	-2.631	0.004*
**Diastolic BP, mmHg**	80.7 ± 10.7	77.1 ± 14.9	-0.962	0.168	78.4 ± 10.7	76.9 ± 7.5	-0.486	0.344
**Glucose, mg/dL**	110.9 ± 67.3	85.8 ± 14.6	-1.083	0.279	116.8 ± 35	97.8 ± 27.8	-2.552	0.011*
**Triglycerides, mg/dL**	172.1 ± 84.3	146.7 ± 71	-0.464	0.650	147.3 ± 72.8	131.3 ± 51.5	-0.804	0.421
**Cholesterol total, mg/dL**	220.8 ± 54.1	212.3 ± 52.8	-0.035	0.972	203.5 ± 56.2	193.3 ± 30.1	-0.393	0.695
**LDL-cholesterol, mg/dL**	136 ± 30.6	136.3 ± 42.9	-0.175	0.861	123.5 ± 48.6	117.4 ± 24.8	-0.175	0.861
**HDL-cholesterol, mg/dL**	42.2 ± 6.2	46.5 ± 13	0.877	0.381	50.9 ± 8.8	49.5 ± 9.3	-0.350	0.727

Values in cells are mean ± standard deviation; **^a ^**p-value for difference in values before and after intervention in Groups 1 and 2 by the Wilcoxon test; *p < 0.05.

## Discussion

The educative interventions - health education workshops and home visits - achieved positive results in the knowledge and grasp of individuals with hypertension regarding the disease and risk factors. There was an increase in knowledge on the disease in both intervention groups, especially in Group 2, thereby demonstrating the greater effectiveness of home orientation. These results underscore the importance of follow up and dialogic, participative health education, considering the low level of schooling of the participants and unsatisfactory knowledge of this population regarding their own health status prior to the intervention.

The acquisition of knowledge about the disease, and ways to control it, favors adoption of attitudes that can influence health at family and community level (25). Health awareness is preceded by full access to information and education as a way to make individuals and communities capable of greater control over their own wellness, which is an essential goal in public health, especially on the primary health care level (7,10,26).

In Group 2, the home orientation strategy - privileged intervention locus - led to beneficial changes in diet, with an increase in the consumption of foods considered either protective or without health risk, especially the consumption of whole-grain cereals. In Group 1, which only underwent the health education workshops, there was also a tendency toward better eating habits, as there was a reduction in the intake of risk foods and an increase in the intake of protective foods, even though there was no statistically significant difference in the consumption of the different food groups before and after the intervention.

The home orientation strategy had a more significant effect on the reduction in oil and sugar intake. As the intake estimates of these foods were calculated based on monthly availability in the residence, one may suppose that the home orientation led to an important alteration in intake not only for the women studied, but also for their families, which demonstrates the importance of home visits in the context of the FHP.

The reduction in salt intake was not statistically significant in either intervention group. A number of studies in the literature indicate that, despite the understanding of individuals regarding the association between salt intake and high blood pressure, this dietary change is not achieved easily (7,26,27,28). However, in the group that received home orientation, those individuals who had an initial salt intake greater than 15 g/day achieved a significant reduction in this parameter by the end of five-month period, thereby demonstrating the positive impact on the home intervention to correct large deviations in salt intake.

The greater adherence of group 2 was confirmed by changes in clinical and nutritional variables. Group 2 achieved a significant reduction in weight, BMI, waist circumference, systolic BP and glucose, whereas Group 1 only achieved a reduction in waist circumference. However, the reduction in waist circumference in Group 1 indicates significant and important dietary modifications, since the abdominal obesity is related to an increased risk of myocardial infarction, stroke and premature death (29). It should be stressed that the educative workshops Group 1 underwent were held with the participants actively engaged in a participative, dialogic, interactive process.

It was not possible to establish a statistical association between dietary changes and alterations in the clinical and biochemical parameters assessed in the different intervention groups. The increase in protective foods - rich in fiber, vitamins and minerals and poor in fat and salt - may at least partially explain the positive results in the clinical, anthropometric and biochemical variables in the group that received home orientation, as demonstrated in studies relating eating patterns and risk factors for cardiovascular disease (30,31).

The home visit, in the context of health education, is an important tool in the consolidation of new practices encouraging the adoption of healthy lifestyles through health promotion and prevention of diseases and disorders. In this sense, the visit allows the understanding of psycho-affective-social and biological characteristics of individuals and families, recognizing the family activity as a privileged locus for interventions. The diagnosis of the home visit prioritizes the individual's reality and education initiatives. It is a key instrument of intervention in family health and continuity of care, being programmed and used in order to support interventions and to provide comprehensive care for individuals and families. It brings a new meaning in the practices of the professionals of the FHP allowing that the socio-economic and cultural surrounding habits, customs and beliefs are considered in their approach. The challenge is to incorporate the home visit as an individual, family and community activity aimed at solving real life problems. In addition, home visit is an important strategy to motivate the individual, family and community to participate in the planning, organization and control of the therapeutic project.

However, the management of home visits in a compulsory manner by health professionals presents serious operational problems. The first concerns the optimization of resources available for health care. The high number of registered families and the high demand for services in the Unit of Primary Health Care makes it impossible to respond to all families through the home visit. Associated with that, are also the work schedules and chores, difficulties related to time and way of locomotion of the team to the households (long walking distances and steep terrain, rain, excessive heat), and fear and ignorance of the population about the purpose of home visit that can disable and/or impair this kind of visit.

Despite the individuals in Group I have demonstrated knowledge acquisition on hypertension and their ways of controlling this alone was not sufficient to promote changes and adoption of the dietary modifications recommended for hypertension. It has been demonstrated that knowledge regarding hypertension does not necessarily imply a change in behavior (7,25).

While reinforcing this in the literature, that points out to the advantages of home visits, especially in regard to the approximation of the health service, represented by the technician, with the reality of family life. It is noteworthy that these results are preliminary and punctual, based on a limited sample, which has major limitations for further generalization of data. This limitation relates mainly to large cities, given the disproportion between the number of cities participating in the program and the enrolled population, generated by the difficulty to expand the Units of Primary Health Care in large cities that face the challenges inherent to the health sector, in addition to the problems posed by urban complexity, such as violence.

## Conclusions

The positive changes in clinical and dietary parameters, statistically higher in the group receiving guidance at home, indicates better adherence to dietary treatment. The results indicate the potential of this strategy in health education as an important tool to improve adherence to non-pharmacological treatment of patients with hypertension. Due to the complexity of dietary behavior, guidance in the household was the most effective strategy in health education and nutrition. It enabled a deeper knowledge of social and cultural dynamics of feeding and interrelation of families, favoring a more appropriate, in the home, and a consequent assimilation of the guidelines by women and families in the actual environment.

Further studies are needed to explore the advantages and limitations of this strategy in contexts of primary health care where the prevalence of hypertension is high.

## Competing interests

The authors declare that they have no competing interests.

## Authors' contributions

AGR - conceived the study, participated in the research literature, methodology, data collection, analysis, discussion of data, drafting the article, and read and approved the final manuscript; RMMC - conceived the study, participated in the process of literature review, methodology, data collection, analysis and discussion of data, drafting the article, directed and coordinated the work, and read and approved the final manuscript.

SMR, CMGCD, AQR, FAFC, MMSV- participated in the analysis, discussion of the data, correcting of the article, and read and approved the final manuscript.

## Pre-publication history

The pre-publication history for this paper can be accessed here:

http://www.biomedcentral.com/1471-2458/11/637/prepub

## References

[B1] World Health Organization40th session of the subcommittee on planning and programming of the executive committee. Regional strategy on an integrated approach to the prevention and control of chronic diseases, including diet, physical activity, and health 2006 Washington: WHO

[B2] Brasil. Ministério da Saúde Hipertensão Arterial Sistêmica 2006 Brasília: MS MS. Normas e Manuais Técnicos

[B3] Ribeiro AG Cotta RMM Ribeiro SMR A Promoção da Saúde e a Prevenção Integrada dos Fatores de Risco para Doenças Cardiovasculares Ciênc Saúde Coletiva [online] 2009 0802 1 1621830317

[B4] Brasil. Ministério da Saúde. Secretaria de Atenção à Saúde. Departamento de Atenção Básica. Coordenação Geral da Política de Alimentação e Nutrição. Guia Alimentar da População Brasileira Promovendo a alimentação saudável 2005 Brasília: MS

[B5] World Health Organization The Global Strategy on Diet, Physical Activity and Health 2003 Washington: WHO

[B6] Starfield B Shi L Macinko J Contribution of Primary Care to Health Systems and Health The Milbank Quarterly 2005 83 3 457 502 10.1111/j.1468-0009.2005.00409.x 16202000 PMC2690145

[B7] Blasco PG Levites MR Janaudis MA Family Medicine Education in Brazil: Challenges, Opportunities, and Innovations Academic Medicine 2008 83 7 684 690 10.1097/ACM.0b013e3181782a67 18580090

[B8] Allegrant JP A Review and Synthesis of Research Evidence for Self-Efficacy-Enhancing Interventions for Reducing Chronic Disability: Implications for Health Education Practice (Part II) Health Promotion Practice 2005 6 2 148 156 10.1177/1524839904266792 15855284

[B9] World Health Organization Adherence to long term therapies: evidence for action 2003 Geneva: WHO

[B10] Kaona ADF Tuba M Siziya S Sikaona L An assessment of factors contributing to treatment adherence and knowledge of TB transmission among patients on TB treatment BMC Public Health 2004 4 68 10.1186/1471-2458-4-68 15625004 PMC545081

[B11] Bodenheimer T Wagner HE Grumbach K Improving Primary Care for Patients With Chronic Illness JAMA 2002 288 15 1909 1914 10.1001/jama.288.15.1909 12377092

[B12] Travassos C Viscava F Pinheiro R Brito A Utilização dos serviços de saúde no Brasil: gênero, características familiares e condição social Rev Panam Salud Publica 2002 11 5/6 365 73 12162833 10.1590/s1020-49892002000500011

[B13] Dias G Franceschini SCC Reis JR Reis RS Batista RS Cotta RMM A vida nos olhos, o coração nas mãos: concepções e representações femininas do processo saúde-doença Hist Cienc Saude-Manguinhos 2007 14 3 779 800 18453330 10.1590/s0104-59702007000300006

[B14] Lewington S Clarke R Qizilbash N Peto R Collins R Age-specific relevance of usual blood pressure to vascular mortalility: a meta-analysis of individual data for one million adults in 61 prospective studies Lancet 2002 360 9349 1903 13 12493255 10.1016/s0140-6736(02)11911-8

[B15] Organização Pan Americana de Saúde. Envelhecimento ativouma política de saúde 2005 Brasília: WHO/OPAS

[B16] Jelliffe DBI Evaluación del estado de nutrición de la comunidad 1968 Genebra: WHO

[B17] Pickering TG Hall JE Lawrence JA Recommendation for blood pressure measurement in humans and experimental animals Part 1: Blood pressure measurement in humans. A statement for professionals from the subcommittee of professional and public education of the American Heart Association Council on High Blood Pressure Research. Circulation 2005 45 142 161 10.1161/01.HYP.0000150859.47929.8e 15611362

[B18] Ribeiro AC Sávio KEO Rodrigues MLCF Costa THM Schmitz BAS Validação de um questionário de freqüência de consumo alimentar para população adulta Rev Nutr 2006 19 5 553 562 10.1590/S1415-52732006000500003

[B19] Siegel S Castellan JR John N Nonparametric Statistics for The Behavioral Sciences 1988 2 New York: McGRAW HILL 399

[B20] Fornés NS Martins IS Velásquez-Meléndez G Latorre MRDO Escores de consumo alimentar e níveis lipêmicos em população de São Paulo, Brasil Rev Saúde Pública 2002 36 1 12 18 11887224 10.1590/s0034-89102002000100003

[B21] Marshall A eBaumann A The internacional physical activity questionnaire summary report of the reliability and validity studies Document of IPAQ Excecutive Commite, World Heath Organization 2001

[B22] Philippi ST Latterza AR Cruz ATR Ribeiro SC Pirâmide Alimentar Brasileira: Guia para escolha dos alimentos 12 1 Rev. Nutr., Campinas 65 80

[B23] World Health Organization Report of a Joint FAO/WHO Consultation. Preparation and use of food-based dietary guidelines 1998 Geneva: WHO 9795598

[B24] American Diabetes Association Standards of Medical Care in Diabetes Diabetes Care 2006 29 supl 4 42 10.2337/dc06-0805 17065711

[B25] Aubert L Bovet P Gervasovi JP Rwebogora A Waeber B Paccaud F Knowledge, Attitudes, and Practices on Hypertension in a Country in Epidemiological Transition Hypertension 1998 31 1136 45 9576126 10.1161/01.hyp.31.5.1136

[B26] World Health Organization First International Conference on Health Promotion - Ottawa Charter for Health Promotion 1986 Ottawa: WHO

[B27] Mant D Effectiveness of dietary intervention in general practice The American Journal of Clinical Nutrition 1997 65 suppl 6 1933 38 10.1093/ajcn/65.6.1933S 9174497

[B28] Korhonen MH Litmanen H Rauramaa R Vaisenen SB Niskanen L Uusitupa M Adherence to the salt restriction diet among people with mildly elevated blood pressure Eur J Clin Nutr 1999 53 880 5 10.1038/sj.ejcn.1600869 10557001

[B29] Larsson B Svardsudd K Welin L Wilhelmsen L Bjorntorp P Tibblin G Abdominal adipose tissue distribution, obesity, and risk of cardiovascular disease and death: 13 year follow up of participants in the study of men born in 1913 BMJ 1984 288 1401 4 10.1136/bmj.288.6428.1401 6426576 PMC1441047

[B30] Champagne CM Dietary interventions on blood pressure: the Dietary Approaches to Stop Hypertension (DASH) trials Nutr Rev 2006 64 suppl 53 56 16532899 10.1111/j.1753-4887.2006.tb00234.x

[B31] Vollmer WM Sacks FM Ard J Appel LJ Bray GA Simons-Morton DG Conlin PR Svetkey LP Erlinger TP Moore TJ Karanja N Effects of diet and sodium intake on blood pressure: subgroup analysis of the DASH-sodium trial Ann Intern Med 2001 135 1019 28 11747380 10.7326/0003-4819-135-12-200112180-00005

[B32] Perera R Heneghan C Yudkin P Graphical method for depicting randomised trials of complex interventions BMJ 2007 334 127 10.1136/bmj.39045.396817.68 PMC1779898 17235093

